# Experimental cell models of insulin resistance: overview and appraisal

**DOI:** 10.3389/fendo.2024.1469565

**Published:** 2024-12-19

**Authors:** Ying Yang, Ting-ting Wang, Hu-ai Xie, Ping Ping Hu, Pan Li

**Affiliations:** ^1^ College of Pharmacy, Chongqing Medical University, Chongqing, China; ^2^ Chongqing Key Research Laboratory for Drug Metabolism, Chongqing Medical University, Chongqing, China

**Keywords:** insulin resistance, type 2 diabetes mellitus, cell model, establishment and evaluation methods, drug screening

## Abstract

Insulin resistance, a key factor in the development of type 2 diabetes mellitus (T2DM), is defined as a defect in insulin-mediated control of glucose metabolism in tissues such as liver, fat and muscle. Insulin resistance is a driving force behind various metabolic diseases, such as T2DM, hyperlipidemia, hypertension, coronary heart disease and fatty liver. Therefore, improving insulin sensitivity can be considered as an effective strategy for the prevention and treatment of these complex metabolic diseases. Cell-based models are extensively employed for the study of pathological mechanisms and drug screening, particularly in relation to insulin resistance in T2DM. Currently, numerous methods are available for the establishment of *in vitro* insulin resistance models, a comprehensive review of these models is required and can serve as an excellent introduction or understanding for researchers undertaking studies in this filed. This review examines and discusses the primary methods for establishing and evaluating insulin resistance cell models. Furthermore, it highlights key issues and suggestions on cell selection, establishment, evaluation and drug screening of insulin resistance, thereby providing valuable references for the future research efforts.

## 1 Introduction

Insulin resistance, defined as a defect in insulin-mediated control of glucose metabolism in tissues such as liver, fat and muscle, is a significant contributor to the development of type 2 diabetes mellitus (T2DM) and cardiovascular disease, particularly in the context of obesity and metabolic syndrome (1–3). Nowadays, it is now widely recognized that insulin resistance is typically characterized by impaired GLUT4 function in muscle and adipose tissue, as well as an inability to suppress hepatic glucose output (1, 4). In conditions of insulin resistance, the release of glucose from the liver (hepatic glucose output), and glucose uptake/utilization into muscle and fat (where it is stored as glycogen) are both inhibited, even in the presence of normal or elevated levels of both exogenous and endogenous insulin (4–6).

**Figure 1:**
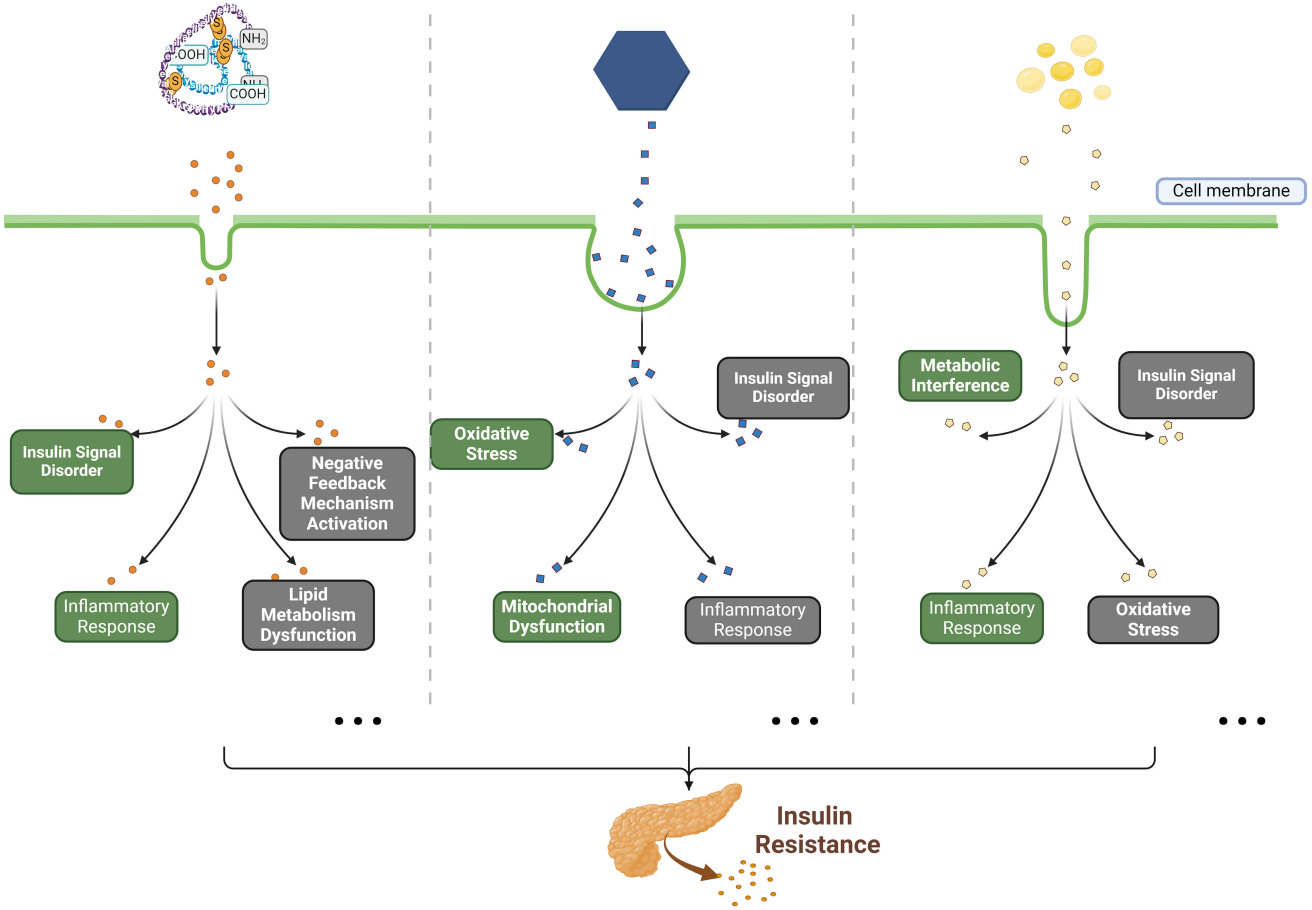
The classical pathogenesis of insulin resistance caused by HCI, HCG and FFAs. Briefly, in previous studies, high concentrations of insulin (HCI), glucose (HCG) and free fatty acids (FFAs) have been identified as the primary substrates for inducing *in vitro* insulin resistance. Mechanistically, HCI disrupts insulin signaling by activating negative feedback mechanisms, impairing lipid metabolism, as well as inducing inflammatory responses; HCG interferes with insulin signaling and impairs glucose metabolism through inducing oxidative stress, mitochondrial dysfunction, and inflammatory responses; FFAs (such as palmitic acid) affect glucose metabolism by causing metabolic disturbances and dysfunction of insulin signaling, while also promoting oxidative stress and inflammatory responses.

Up to now, a multitude of potential pathogenic factors and the related pathogenesis of insulin resistance have been proposed, including genetics, obesity, aging, exercise, diet, etc. (7–9). Among these factors, diet-induced metabolic dysfunction plays a significant role in the development of insulin resistance currently (10). Diets high in calories, characterized by an excessive intake of carbohydrates and fats and a low dietary fiber consumption, can predispose individuals to insulin resistance (11, 12). Mechanistically, several mainstream or classical pathological mechanisms of insulin resistance have gained widespread recognition, including oxidative stress, inflammatory response, insulin signal disorder, endoplasmic reticulum stress, as well as mitochondrial dysfunction (13–15). These conditions can be induced by the excessive intake of carbohydrates (such as glucose) or fats (especially for saturated fatty acids), the chronic insulin exposure, and the inflammatory factors such as tumor necrosis factor alpha (TNF-α) (11–13, 16, 17). The representative mechanisms of insulin resistance have been described in **Figure 1** (mainly including insulin resistance caused by high glucose, high insulin and free fatty acids). In recent years, the pathogenesis of insulin resistance has remained a hot issue in metabolic disease research. Notably, the studies by James and colleagues have demonstrated that mitochondrial oxidative stress can cause insulin resistance without disrupting oxidative phosphorylation, and the elevated mitochondrial ceramide levels augment mitochondrial membrane permeability and apoptosis, leading to insulin resistance (15, 18, 19). In addition, growing evidence suggests a close relationship between gut microbial dysbiosis and insulin resistance, as is found that individuals with low bacterial gene counts exhibited higher insulin resistance, dyslipidemia, and inflammation compared to those with higher bacterial gene counts (20, 21). Taken together, these findings provide new insights into the pathogenesis of insulin resistance.

Nevertheless, the molecular mechanisms contributing to insulin resistance still remain incompletely understood, and it is critical and necessary to establish insulin resistance models for mechanism study or drug screening. While animal models, primarily using rats and mice, have been pivotal in insulin resistance research due to its biological resemblance to human, the characteristics of animal study limit its application, such as the lengthy feeding cycles, high costs, complex procedures, and ethical considerations. In contrast, cell culture is highly desirable due to its accessibility and affordability. It serves as a valuable tool for studying mechanisms, conducting bio-activity screening, and assessing toxicity. Cell-based screening studies offer several advantages over animal studies. For instance, it is easier to control due to the fewer influencing factors, including lower individual differences and less impact from environmental or geographical variations, thereby enhancing the reproducibility in the experimental process. Additionally, cell studies are convenient, rapid, and cost-effective, making them an attractive alternative to animal research.

In previous insulin resistance-associated studies (22–24) cell-based investigations have been commonly employed for drug screening and mechanistic exploration. Numerous methods are available for the establishment of *in vitro* insulin resistance models in published data, a comprehensive review of these models is required and can serve as an excellent introduction or understanding for researchers undertaking studies in this filed. Therefore, our current review aims to provide an exhaustive overview and appraisal of the published *in vitro* insulin resistance models. Meanwhile, this review also presents guidelines for the comprehensive process of constructing these cell models, including the selection of cell lines, the induction conditions, and the evaluation methods. Such comprehensive guidance may serve as a valuable reference for future research in this area.

## 2 Data sources

We conducted a literature search using databases such as PUBMED, GOOGLE SCHOLAR, and WEB of SCIENCE, utilizing keywords such as “insulin resistance” or “insulin resistance and cell models”. Subsequently, we systematically reviewed and summarized the literature retrieved from these databases (up to 1 Nov 2024).

## 3 Overview and appraisal

### 3.1 Cell lines for insulin resistance

The liver, muscle and adipose tissue are universally acknowledged as the primary tissues sensitive to insulin. Consequently, *in vitro* models of insulin resistance are typically generated using cell lines derived from these tissues. This review provides a comprehensive summary of the cell lines utilized, with an in-depth elaboration on each.

#### 3.1.1 Liver

##### 3.1.1.1 HepG2 cell

The human hepatoma cell line, HepG2, is a widely utilized model for hepatic cells and has been employed in diverse research areas ranging from oncogenesis to the investigation of substance-induced liver phenotypes. Originating from human liver cancer, HepG2 cells present numerous typical characteristics of liver cells, such as the ability to express a variety of liver-related enzymes and transport proteins. Concurrently, HepG2 cell line maintains the insulin response mechanisms of hepatocytes, making it as an ideal model for studying insulin resistance in liver cells (25, 26).

Over the past decade, a review of published references reveals that the HepG2 cell line is the most commonly used model for studying the pathological mechanisms and conducting drug screening related to insulin resistance (a total of 1381 papers in PUBMED searching, using the keywords “HepG2 cells, insulin resistance”). For instance, studies using HepG2 cells have demonstrated that early growth response proteins-2 (Egr2) enhances insulin resistance via JAK2/STAT3/SOCS-1 pathway (27), PKM2 may promote hepatic insulin resistance via STAT3 pathway (28), and ASGR1 is a potential intervention target for improving systemic insulin resistance (29). Furthermore, the HepG2 cell line is increasingly being used in drug screening for insulin resistance (30–32), with some studies exclusively employing it as an *in vitro* model (33–35).

Despite the HepG2 cell line becoming a widely utilized choice in research due to its accessibility, robust adaptability and stability *in vitro*, it presents certain limitations when employed for insulin resistance studies. Originating from tumor cells, HepG2 cells show distinct characteristics compared to normal liver cells, which may lead to abnormal activation of signal pathways or gene mutations, thus hindering the establishment of accurate insulin resistance models. For example, the inhibition of FOXK1 expression in hepatocellular carcinoma cells reduces cellular aerobic glycolysis and cell viability by impeding the transduction of the AKT/mTOR signaling pathway, a function distinct from that of Foxk1/2 in normal liver cells (36). Alterations in the transforming growth factor-beta (TGF-β) signaling pathway in HepG2 cells may influence inflammation, fibrogenesis, and immunomodulation compared to normal liver cells (37, 38). Furthermore, HepG2 cells are less differentiated than primary hepatocytes, lacking some typical hepatocyte functions and metabolic characteristics, which can exacerbate the probing defects in HepG2 cells (such as showing a weaker insulin sensitivity than primary hepatocytes) (39–41). These factors should be carefully considered when selecting HepG2 cell line, especially for studying pharmacological mechanisms. Additionally, the basal expression or activity of these genes in HepG2 cells should be taken into account in gene knockdown or overexpression experiments.

##### 3.1.1.2 L02 cell

The human hepatocyte L02 cells (L02) are also frequently employed in the development of hepatic insulin resistance models *in vitro* (a total of 43 papers in PUBMED searching, using the keywords “L02 cells, insulin resistance”). As a normal human liver cell line, L02 cells bear closer resemblance to actual liver cells in terms of cellular characteristics and functions, including metabolic pathways, physiological enzyme levels and gene expression patterns. Hence using L02 cell line can accurately mirror the mechanisms of hepatocyte insulin resistance under both physiological and pathological conditions (42, 43). Meanwhile, a study indicates that L02 cell line shows a slightly narrower toxicity threshold (assessed by CCK8 assay), making it more stable and sensitive than HepG2 cells in assessing drug-induced liver injury (44). In previous studies, L02 cell line has been used to validate that curcumin ameliorates bisphenol A-induced insulin resistance by inhibiting the JNK pathway (45), to demonstrate that mitochondrial oxidative stress-induced hepatic insulin resistance can be mitigated by sphingosine 1-phosphate (46), as well as to assess the effects and mechanism of substances such as pterostilbene and Annexin A1 on hepatic insulin resistance (47, 48). Furthermore, we observed that L02 and HepG2 cell models are frequently used in conjunction for the activity screening of anti-insulin resistance (49–51).

We also note that, the establishment of an insulin resistance model *in vitro* using L02 cells may take longer time compared to HepG2 cells, and regardless of whether it is induced by HCI, FFAs or other agents, the induction period typically spans 24-72 h (49, 51, 52). Meanwhile, based on our experience and feedback from other research groups, L02 cells are highly sensitive to environmental factors such as temperature, composition of the culture medium and pH levels. In addition, L02 cell line shows a slower growth rate compared to HepG2 cells.

##### 3.1.1.3 Primary hepatocyte

Primary hepatocytes are typically procured from the livers of animals, such as rats and mice, due to the scarcity of human liver tissue and ethical considerations. These cells are extracted directly from liver tissues, offering superior physiological relevance, robust metabolizing enzyme activity and validated gene expression in comparison to cell lines (53–56). Primary hepatocytes are often employed in conjunction with *in vivo* insulin resistance models to explore the pathogenesis of insulin resistance, screen for drug activity, and elucidate pharmacological mechanisms (a total of 656 papers in PUBMED searching, using the keywords “primary hepatocytes, insulin resistance”). For instance, primary hepatocytes have been employed to assess the impact of Inositol polyphosphate multikinase on FFAs-induced insulin resistance (57), to evaluate the role of chemotactic eicosanoid LTB4 in dexamethasone-induced insulin resistance (58), as well as to determine the insulin sensitization effect of short-term curcumin gavage in a rapid dexamethasone-induced insulin resistance (59).

Despite the recognized benefits of employing primary hepatocytes, their application appears to be less prevalent than that of hepatic cell lines, particularly in pharmacological mechanism investigations. This discrepancy may be ascribed to the complexities involved in obtaining and culturing primary hepatocytes, as well as the inherent variability derived from different individual sources, which consequently affects the high reproducibility. Additionally, primary cells from animals (such as rats or mice) have species-specific physiological differences compared with human cells, making it difficult to extrapolate the results to humans.

#### 3.1.2 Muscle

##### 3.1.2.1 C2C12 cell

The C2C12 cell line is derived from mouse myoblasts and has the capacity to differentiate into mature muscle tubes under specific culture conditions. C2C12 cells exhibit physiological characteristics akin to skeletal muscle cells, including the expression of myosin heavy chains and the formation of sarcomeres. Thus it has been utilized extensively as an *in vitro* model in understanding metabolic disease progression, including insulin resistance (60). In previous studies, C2C12 cells appear to be the most frequently used cell type for *in vitro* construction of muscle insulin resistance models (a total of 666 papers in PUBMED searching, using the key words “C2C12 cells, insulin resistance”). The findings that mangiferin ameliorates insulin resistance by activating the PPARα pathway (61), resveratrol enhances PA-induced insulin resistance via the DDIT4/mTOR pathway (62), and downregulation of lipin-1 induces insulin resistance by elevating intracellular ceramide accumulation (63), were all derived from the experiments using insulin resistance C2C12 cell models.

A comprehensive review of studies on the C2C12 cell line underscores its considerable importance in pharmaceutical and biomedical research, attributable to its expression of glucose transporter (GLUT)-4 and other characteristics that bear a striking resemblance to those of human skeletal muscle cells (60). While it is acknowledged that C2C12 cells possess the capability to differentiate into myotubes, it is imperative to recognize that their differentiation might not be entirely analogous to that of actual skeletal muscle cells. Incompletely differentiated cells may manifest diminished GLUT4 expression, frequently in conjunction with modifications in the expression or activity of pivotal proteins within the insulin signaling pathway (64, 65). Considering that glucose uptake serves as a “gold standard” for evaluating insulin resistance during drug screening, C2C12 cells might present certain limitations due to their suboptimal responsiveness to insulin-stimulated glucose uptake, thereby necessitating the employment of L6 cells as an alternative (66). Additionally, the C2C12 cell line demonstrates resistance to both gene transfection and viral transduction, thereby constraining its application in pertinent cellular molecular biology research (67).

##### 3.1.2.2 L-6 myoblast

L-6 myoblasts (L6 cells), a type of rat skeletal muscle myoblast cell, are extensively utilized in research pertaining to muscle development, diseases, and metabolic disorders. Their capacity to differentiate into mature myotubes under controlled *in vitro* conditions, coupled with their robust insulin sensitivity, makes them ideal for constructing *in vitro* models of muscle insulin resistance (68, 69). In previous studies (a total of 477 papers in PUBMED searching, using the key words “L6 cells, insulin resistance”), L6 cells have been employed to explore the role of NF-κB activation in the development of insulin resistance (70), to study the mechanism of HM-chromanone in alleviating obesity-related insulin resistance (71), and to assess the impact of metformin on attenuating insulin resistance (72).

Skeletal muscle serves as a crucial target tissue for insulin action within the body, playing an instrumental role in the regulation of blood glucose. L6 cells, which serve as a model for skeletal muscle cells, are intimately associated with the mechanism of insulin resistance. L6 cell line can effectively simulate the response of skeletal muscle cells to insulin *in vivo*. It also exhibits a GLUT expression profile similar to fully differentiated mammalian muscle cells, demonstrating high levels of GLUT4 (the sole insulin-dependent glucose transporter in skeletal muscle) and comparatively low expression of GLUT1 and GLUT3, which results in a low basal glucose uptake rate but a pronounced response to insulin stimulation (66, 73). Furthermore, the highest expression level of GLUT4 is observed in L6 cells stimulated with insulin, surpassing that of C2C12 and HMSC cells (66). And meanwhile, the sensitivity of mitochondrial respiration in L6 cells to mitochondrial poisons is analogous to that observed in primary muscle fibers, indicating that the L6 cells embody the requisite metabolic characteristics necessary for exploring the role of mitochondria in insulin resistance (74). Taken together, these evidence suggest that L6 cells show a heightened sensitivity to insulin.

Although both C2C12 and L6 cells have been frequently utilized in previous studies to establish insulin resistance models *in vitro*, a recent study using transcriptomics and metabolomics highlights significant differences between the two different skeletal muscle models. It is found that the L6 cells show the highest expression levels of glucose transporter and mitochondrial electron transfer genes, coupled with superior insulin-stimulated glucose uptake and oxidation capacity, which renders L6 cells particularly suitable for investigations into glucose metabolism and mitochondrial function (66). Conversely, C2C12 cells with mRNAs encoding for actin and myosin are significantly enriched, have higher glucose oxidation capacity than L6 cells; meanwhile, C2C12 cell line shows a similar to the differentiated muscle tissue in myofibrillar content and glycogen storage, making it more suitable for exercise/stress response studies (66). In addition, the mechanisms through which FFAs induce insulin resistance differ between these two cell types. For example, evidence suggests that the myocellular abundance of caveolin-enriched domains (CEM) located at the plasma membrane is significantly higher in L6 cells compared to C2C12 cells, which leads to the distinct mechanisms of “PA-ceramide”-induced insulin resistance observed in both cell types (ectopic accumulation of ceramide in response to oversupply of PA may underlie the development of insulin resistance in skeletal muscle) (75–77). Taken together, our present understanding suggests that L6 cells may be a more appropriate cell line for the establishment of skeletal muscle insulin resistance *in vitro*. However, the differences of utilizing these two cell types for the construction of an insulin-resistant cell model, particularly in relation to functionality and underlying mechanisms, still need further exploration.

#### 3.1.3 Adipose

##### 3.1.3.1 3T3-L1 Cell Line

The 3T3-L1 cell line, a well-established preadipose cell line derived from murine Swiss 3T3 cells, is frequently employed in studies investigating adipocyte differentiation and insulin resistance (78, 79). Upon full differentiation, 3T3-L1 cells display essential characteristics of mature adipocytes, including lipid accumulation and the expression of adipogenic transcription factors such as PPARγ and C/EBPα (79–81). Adipocytes play a crucial role in glucose uptake and insulin response, which contributes to the progression towards insulin resistance (82–84).

3T3-L1 cells are the most commonly used cell model for adipocyte insulin resistance research, including basic molecular mechanism studies and drug screening (2151 papers in PUBMED searching using “3T3-L1 cells, insulin resistance” as key words). In recent years, 3T3-L1 cells are employed in studies about insulin resistance, such as assessing the effect of phenethyl isothiocyanate on H2O2-induced insulin resistance (85), exploring the role of endogenous CSE/H2 S system in TNF-α-induced insulin resistance (86), and evaluating the activities and mechanisms of natural products (geniposide or baicalin) in improving insulin resistance (87, 88). Notably, comparing to the freshly isolated cells, 3T3-L1 cell line is easier to culture and can tolerate a greater number of passages and offers a homogeneous cell population, which exhibits a homogenous response following treatments and changes in experimental conditions (79, 89). However, the 3T3-L1 cell model has some limitations in establishing insulin resistance. For example, the initial subculture necessitates a minimum duration of two weeks for adipogenic differentiation (90). Moreover, when confluent or extensively passaged, 3T3-L1 cells lose their ability to differentiate into adipocytes and become difficult to transfect (79, 91).

##### 3.1.3.2 SGBS Cell Line

The Simpson-Golabi-Behmel syndrome cell line (SGBS) is a human cell model derived from preadipocytes of a patient diagnosed with Simpson-Golabi-Behmel syndrome, and it has been extensively utilized in research to study adipocyte biology and metabolic functions (82, 92). Initially isolated from subcutaneous adipose tissue, SGBS cells demonstrate a high capacity for adipogenic differentiation and exhibit key characteristics (such as gene expression profile and metabolic functionality) of mature adipocytes (including primary human adipocytes) (82, 93, 94). Therefore, SGBS cell lie serves as an ideal model for investigating human adipocyte differentiation, insulin resistance, and metabolic disorders. In recent years, SGBS cells have been applied to insulin resistance research (a total of 41 papers in PUBMED searching, using the key words “SGBS cells, insulin resistance”). By using SGBS cells model, it was found that N-Methylpyridinium can attenuate TNF-α-mediated insulin resistance and inflammation (95), Interferon-gamma alters adipocyte phenotype and impairs response to insulin and adiponectin release (96), and miR-146a regulates systemic and adipocyte insulin sensitivity via downregulation of NPR3 (97). However, SGBS cells also present limitations in establishing *in vitro* models of insulin resistance. The growth and differentiation capacity of SGBS cells in long-term culture can be easily affected by the environmental factors such as oxygen concentration and culture medium composition, which may potentially compromise the reproducibility and complexity of the experiments (98). Additionally, in contrast to primary cells, SGBS cells show a reduced sensitivity to apoptosis-inducing stimuli under certain conditions, which may limit their effectiveness in studying adipocyte biology under stress (98).

Overall, the 3T3-L1 and SGBS cell lines are the mainly used adipocytes for establishing *in vitro* cell models. However, several considerations should be taken into account when deciding between these two cell lines. In chronic insulin stimulated-insulin resistance model, the basal and insulin-stimulated glucose uptake is more profoundly affected in insulin resistant 3T3-L1 cells compared to SGBS cells (82). While both cell lines can be induced to insulin resistance by using FFAs, insulin, and TNF-α (discussed further in the subsequent sections), a study indicates that SGBS cell line is more sensitive than 3T3-L1 to insulin signaling disruptions induced by FFAs such as PA, because it shows a more pronounced recovery of Akt phosphorylation in response to insulin stimulation and a closer resemblance to human primary cells in signaling responses (99). In addition, studies suggest that, fully differentiated SGBS cells, rather than 3T3-L1 cells, present a more similar morphology, transcript level, and biochemical function to the primary omental adipocytes associated with obesity-induced metabolic disorder (79, 100, 101). Interestingly, it is found that SGBS cells not only show white adipocyte functionality but also possess the ability to differentiate into brown or beige adipocytes, providing a valuable platform for exploring the browning process in human fat tissue (82).

### 3.2 Induction conditions for insulin resistance

In previous studies, the most commonly used methods for inducing insulin resistance include high concentrations of insulin (HCI) (102, 103), high concentrations of glucose (HCG) (104, 105), and high levels of FFAs (106, 107), alongside other inducers like dexamethasone (108, 109), glucosamine (GlcN) (110, 111), uric acid (UA) (112), and pro-inflammatory cytokines such as tumor necrosis factor-α (TNF-α) (113, 114). Each method has its unique advantages and limitations in replicating various aspects of insulin resistance. In this Review, we primarily focus on the three most commonly used substrates, including HCI, HCG and FFAs, which are frequently employed to establish *in vitro* insulin resistance in recent publications.

#### 3.2.1 High concentration of insulin

Using HCI to impair insulin signaling and cause insulin transduction dysfunction *in vitro* is a generally employed method for establishing insulin resistance cell model (82, 115, 116). Mechanistically, HCI triggers the preferential internalization and degradation of kinase-competent insulin receptors, resulting in a population of receptors with multiple functional abnormalities accumulated on the cell surface. And this process reduces the number of insulin receptors, with the extent of reduction directly proportional to the levels of insulin and the duration of stimulation (117, 118). Hepatic cells, primarily HepG2 cells (119, 120), adipocytes such as 3T3-L1 cells, SGBS cells and primary adipocytes (121–125), as well as muscle cells like C2C12 (126, 127), are frequently employed to establish an insulin resistance model via HCI *in vitro*. In these studies, exposure times typically range from 24 to 48 hours and concentrations vary from 100 to 1,000 nM. These conditions have been documented to decrease glucose uptake and downregulate or inhibit key insulin signaling proteins such as IRS1 and GLUTs (82, 115, 122, 128–130).

Chronic hyperinsulinemia is a primary contributor to the exacerbation and initiation of insulin resistance, leading to the development of overt T2DM. Numerous studies have demonstrated the efficacy of chronic insulin exposure in the induction of insulin resistance models *in vitro* (82). In previous studies, Fan et al. employed a concentration of 100 nM insulin for 36h to induce insulin resistance and studied the beneficial effect of ginsenoside-Rg1 on glucose metabolism in HepG2 cells (129). Similarly, Vlavcheski et al. exposed L6 cells to 100 nM insulin for 24h to induce insulin resistance cell model and explored the potential effect of resveratrol against insulin resistance in muscle cells (115). Nevertheless, there are still many limitations in HCI induction. For example, HCI induction does not fully replicate the multifactorial nature of insulin resistance *in vivo*, where additional factors like lipotoxicity and inflammation also play a significant role (131). Meanwhile, evidence indicates that the effect triggered by HCI may be temporary, with cells potentially reverting to their original insulin sensitivity levels upon cessation of the insulin treatment, which poses challenges for conducting long-term studies or experiments that necessitate a consistent state of insulin resistance (132, 133). Furthermore, under physiological conditions, insulin secretion is meticulously regulated, dynamically adjusting in response to various factors such as blood glucose levels (134, 135). However, the HCI observed *in vitro* significantly deviates from physiological states in terms of insulin secretion regulation, mechanism of action, and overall metabolic environment, failing to accurately represent the physiological state. In conclusion, these limitations of HCI-based construction of insulin resistance and their induced outcomes should be taken into consideration in future research.

#### 3.2.2 High concentration of glucose

Excessive glucose (relevant for hyperglycemia) can induce serine/threonine phosphorylation of IRS protein at multiple sites, which impedes the binding affinity of IRS to insulin receptor and suppresses the activation of PI3K/AKT pathway-mediated glucose metabolism (131). Meanwhile, it has been noted that HCG also activates the MAPK and NFκB pathways, both of which play a significant role in the development of insulin resistance (136, 137). Previous studies have used a variety of cell lines, including HepG2 cells, 3T3-L1 cells, L6 cells, and primary hepatocytes, to induce insulin resistance *in vitro* using HCG (35, 138–140). In published data, the induction of insulin resistance in cells is typically achieved by exposing them to glucose concentrations between 25 mM and 60 mM over a period of 24 to 48h (139–143). For instance, Zhang et al. established the insulin resistance model by exposing cells to 25 mM glucose and discovered that Epigallocatechin gallate ameliorated insulin resistance by modulating inflammation and oxidative stress in HepG2 cells (104). Luo et al. treated C2C12 cells with 60 mM glucose for 5 days to explore the underlying mechanisms of HCG-induced insulin resistance (144).

HCG-induced insulin resistance model, replicating the hyperglycemia observed in diabetic patients, is employed to investigate the mechanisms of insulin resistance and anti-insulin resistance drug screening *in vitro*. It is also known that HCG can induce cell damage, called glucose toxicity. Cellular responses to high glucose are numerous, and HCG induction can inflict cellular damage by compromising cell membrane integrity or hastening ROS-mediated stress responses, further impair mitochondrial function, resulting in cellular damage and apoptosis (145, 146). Therefore, cell viability should be considered when constructing an insulin resistance model, particularly for drug screening and evaluation purposes. This aspect is not consistently addressed in published studies, which will be elaborated upon in our subsequent evaluation section. Furthermore, it is significant to ensure that the glucose concentration should be physiologically appropriate. As is described in a few studies, more than 30 mmol/L glucose has been used to represent the hyperglycaemia condition (142, 147, 148). However, we recommend that glucose levels should not exceed 25-30 mmol/L to mimic the diabetic conditions accurately. Levels above 30 mmol/L (600 mg/dL), accompanied by serum osmolality >320 mOsm/kg, induce signs and symptoms related to the hyperosmolar hyperglycemic state. Treating cells with such high glucose concentrations is not pathophysiologically relevant, rendering the findings from these experiments meaningless.

#### 3.2.3 Free fatty acids

FFAs, primarily consisting of PA and oleic acid (OA), are widely recognized as crucial pathogenic factors in insulin resistance and the development of T2DM (42, 149). Although the mechanisms underlying how obesity and FFAs-induced insulin resistance remain incompletely understood, mainstream views suggest that excessive FFAs can lead to dysfunction in gluconeogenesis and glycogen synthesis by increasing the level of intracellular phosphorylated glycogen synthase, down-regulating the phosphorylation of protein kinase B (p-AKT), reducing the expression of glycogen synthase, and inhibiting the insulin signal pathway (132, 150, 151). Concurrently, FFAs-induced oxidative stress and inflammatory response play important roles in insulin resistance development. FFAs produce low-grade inflammation through activation of nuclear factor-kappa B (NF-κB), leading to the release of the pro-inflammatory factors and activation of oxidative stress-activated signaling pathways in the liver and skeletal muscle (152, 153). Therefore, FFAs can also be utilized as inducers for modeling insulin resistance, particularly in replicating obesity-induced insulin resistance *in vitro*.

From previous studies, the induced concentrations of FFA (main PA) typically range from 100 µM to 1,000 µM, with exposure periods spanning between 6 and 48h (106, 107, 154). As is shown that, Wu et al. simulated obesity-induced hepatic insulin resistance using 0.25 mM PA and examined the positive impact of apigenin on alleviating PA-induced insulin resistance in HepG2 cells (106). Lei et al. treated HepG2 cells with 1 mM FFAs (mixture, OA and PA in a ratio of 2:1) for 24 h to induce the insulin resistance model and evaluated the anti-insulin resistance effect of vaccarin (a natural product) (155). Furthermore, FFAs such as OA or linoleate, can also induce insulin resistance; but the mechanisms and effects of this insulin resistance differ from those induced by PA (156). A review of existing literature reveals that PA is more commonly used *in vitro* for the creation of insulin resistance models compared to other free fatty acids such as OA (as was determined by a search of 1082 papers using the keyword “palmitic acid, insulin resistance” in PUBMED and 591 papers using “oleic acid, insulin resistance.” In previous studies, OA appears to facilitate the *in vitro* development of a lipid-accumulating cell model. Interestingly, it was found that low dose of OA or linoleate has a protective effect against PA-induced cytotoxicity and insulin resistance (157–159). Therefore, this protective effect of OA should be noted when using mixed FFAs (such as a mixture of PA and OA) to induce insulin resistance models *in vitro*. Meanwhile, it is suggested that the cell viability should also be considered when evaluating the models of insulin resistance caused by FFAs due to their cytotoxicity (160, 161).

#### 3.2.4 Other inducers

Over the past decades, although the causal relationship between inflammation and insulin resistance is not completely understood, a large number of animal and human studies have strongly suggested that chronic inflammation in adipose tissue plays an important role in the development and progression of obesity-related insulin resistance (162, 163). Pro-inflammatory cytokines such as TNF-α are widely used in insulin resistance cell models. TNF-α is mainly produced by adipocytes and peripheral tissues, which induce localized inflammation through reactive oxygen species (ROS) generation and transcription-mediated signaling pathways. In addition, TNF-α inhibits insulin signaling by increasing serine phosphorylation of IRS-1, thereby reducing the ability of IRS-1 to transmit downstream signals. This inhibition leads to a decrease in GLUT4 translocation and glucose uptake, resulting in insulin resistance (164–166). TNF-α is commonly used at concentrations of 10 to 50 ng/mL for 24 to 48h (147, 167). The advantage of this approach is that it closely mimics the pro-inflammatory environment associated with obesity-related metabolic disorders. However, the broad effects of cytokines can lead to unintended cellular responses, complicating data interpretation.

Uric acid (UA) is another agent associated with insulin resistance, particularly in liver cells (168, 169). Concentrations of UA ranging from 600 to 1,000 µM were applied for 24 to 48h (51, 168). UA-induced insulin resistance models are especially useful for studying the effects of hyperuricemia on insulin signaling, lipid metabolism, and inflammation in hepatic cells. Experimental evidence demonstrates that UA exposure elevates serine phosphorylation of IRS-1 while simultaneously reducing phosphorylation of AKT, resulting in impaired translocation of the glucose transporter GLUT4 and subsequently diminishing cellular glucose uptake (112, 170). Additionally, UA activates the NLRP3 inflammasome and promotes the secretion of pro-inflammatory cytokines, driving chronic inflammatory responses. This sustained inflammation within adipose tissue and other target organs further disrupts insulin signaling pathways, thereby exacerbating insulin resistance (171). However, the use of high concentrations of UA can lead to oxidative stress, potentially compromising cell viability (172).

Glucosamine (GlcN), a precursor of the hexosamine biosynthetic pathway, is known to induce insulin resistance in peripheral tissues and has been utilized extensively for this purpose both *in vivo* and *in vitro* (173, 174). The mechanism by which GlcN induces insulin resistance involves depletion of intracellular ATP, impairment of insulin signaling, and inhibition of GLUT4 translocation (175, 176). Typically, GlcN is employed at concentrations around 18 mM, with exposure durations ranging from 18 to 24 hours (110, 155). While GlcN-induced models are beneficial for investigating the metabolic effects of alterations in glucose metabolism in insulin resistance, it is important to note that the mechanisms underlying GlcN-induced insulin resistance do not entirely align with those induced by hyperglycemia. This is because the two differ in specific metabolic pathways and may not fully replicate the comprehensive pathophysiology of insulin resistance (176, 177).

### 3.3 Evaluation for insulin resistance

In pathological conditions, insulin resistance results in diminished glucose uptake and utilization. This is evident in the decreased glucose intake and glycogen synthesis, as well as the accelerated gluconeogenesis (6, 131). Consequently, phenotypic alterations linked to insulin resistance have been employed to assess insulin resistance models and have become the benchmark for evaluating the efficacy of anti-insulin resistance drugs. Here, we detail the prevalent methods used to evaluate insulin resistance.

#### 3.3.1 Glucose uptake

It is widely recognized that in states of insulin resistance, glucose uptake or utilization in insulin-sensitive tissues such as the liver, muscle, or fat is markedly diminished in response to insulin stimulation. Therefore, the glucose uptake appears to be the “gold standard” for evaluation of insulin resistance or drug screening *in vitro*. In previous studies, two methodologies were employed to detect glucose uptake *in vitro*.

##### 3.3.1.1 Glucose oxidase-peroxidase assay

The glucose oxidase-peroxidase (GOD-POD) assay is widely employed for determining cellular glucose uptake, which is based on Trinder reaction principle (178, 179). Briefly, glucose is converted to gluconic acid and hydrogen peroxide by glucose oxidase (GOD), followed by peroxidase (POD) catalyzing hydrogen peroxide and inducing the formation of quinone imines from the chromogenic material (4-aminoantipyrine). The color intensity of quinone imines is proportional to the glucose concentration, allowing for measurement and calculation via absorbance. As demonstrated in most previous studies, celluar glucose uptake can be calculated with a formula: the glucose uptake equals the difference between the glucose content of the detection solution and the residual glucose content in the medium (128, 180, 181). The GOD-POD assay is characterized by its simplicity and convenient operation, making it the convenient method for studying glucose uptake of cells.

##### 3.3.1.2 Fluorescently labeled glucose

Fluorescently labeled glucose uptake assay, such as2-(N-(7-Nitrobenz-2-oxa-1,3-diazole-4-yl) amino)-2-deoxy-D-glucose (2-NBDG) fluorescently labeled glucose, enables direct monitoring of glucose uptake in living cells and tissue (182, 183). Published data delineate two categories of detection and evaluation methods for monitoring and assessing glucose uptake in cells. The distinction of these two methods lies in the presence or absence of insulin stimulation before measuring glucose uptake. As previous studies described, 100 nM insulin was used to induce IR in HepG2 cells, and in order to evaluate the model or anti-IR activity, a 2-NBDG assay was performed to detect glucose uptake following stimulation of the cells with 100 nM insulin for 30 min (184, 185). Another report introduced an identical method as described above, albeit without insulin stimulation. Instead, it directly detected cellular glucose uptake after exposing them to fluorescent-labeled glucose for 30min (186). Generally, 2NBDG glucose uptake assay is widely used in most studies due to its convenience. However, a recent study has found that the excess glucose nor pharmacological inhibition of GLUT1 impacted 2NBDG uptake in myeloma cells or primary splenocytes, suggesting the 2NBDG uptake in the cellular uptake of 2NBDG may not be a reliable measure of glucose transport, as it is facilitated by an unidentified mechanism (187). Notably, the generalizability of 2NBDG to other tissue cells and the defined mechanisms still need further investigation, which should also be addressed in future studies

In addition, with the development of interdisciplinary fields such as physics, chemistry, and biology, other fluorescent labeled glucose have been also introduced for determining glucose uptake, including FITC-labeled-glucose analog (188), near-infrared fluorescent glucose tracer (Glc-SiR-CO2H) (189), radiolabeled 3-O-methylglucose (190) etc. And these methodologies are referenced by evaluation system of 2-NBDG labeling as described above, and can proficiently oversee glucose uptake in living cells and provide monitoring images.

It is important to note that, according to published data, when it comes to investigating glucose uptake in insulin resistant cells, relative quantification methods are frequently used. The normalization for relative quantification can better reflect the overall glucose uptake of cells with or without treatment. For cell study, it is well-known that cell number or viability plays an important role in the evaluation of metabolic indexes *in vitro*, especially in studies of insulin resistance. However, this factor is not always clearly addressed in previous studies, leading to unnecessary misunderstandings of the results (191–193). As we described, the HCG, HCI, FFAs as well as inflammatory factors such as TNF-α, can impair cell viability and even cause apoptosis in various cell lines (145, 146, 194–196). Without doubt, the glucose uptake is positively correlated with cell number or viability. If the inducers (such as HCI, HCG and FFAs) cause a reduction or increase of cell number, the corresponding glucose uptake/consumption will also change. To eliminate the interference caused by cell apoptosis, the normalization of uptake ratio should be used to evaluate insulin resistance. It can be done using the formula to normalize glucose uptake as described (128, 141, 186): normalization glucose uptake=glucose uptake/cell viability (MTT, CCK8), as MTT or CCK8 can relatively reflect the condition of cells.

##### 3.3.1.3 Radiolabeled glucose

Radiolabeled glucose uptake assays, such as [3H]-2-deoxy-D-glucose or 3-O-[Methyl-3H] glucose, have been used to study glucose metabolism *in vivo* at the molecular level (151, 190, 197–199). Radiolabeling with glucose analogues is the gold standard for measuring glucose uptake, with selectivity for tissue, high cellular uptake, affinity for glucose transporters, retention, and good interaction with hexokinase (200). These analogues are reliable tracers that enter cells through the same transport mechanisms as glucose (201). However, their radioactivity and procedural complexity limit their use, requiring careful experimental design and safety precautions.

#### 3.3.2 GLUT4 translocation assay

GLUT4 is predominantly sequestered in intracellular GLUT4 storage vesicles (GSVs). Insulin initiates the brisk repositioning of GSVs from the trans-Golgi network (TGN) and/or endosomes to the plasma membrane (PM), where fusion occurs, thereby enhancing glucose uptake (202). Studies have shown that overexpression of GLUT4 could improve insulin resistance and insulin action in diabetic mice, indicating that GLUT4 translocation plays a crucial role in whole-body glucose homeostasis (203, 204). Currently, endogenous GLUT4 translocation has been used as an *in vitro* readout, and the widely used methods include immunofluorescence/immunoblotting (analysis of the extracted PM protein) (205, 206) and tracer assay. Among these methods, GLUT4 trafficking assays rely heavily on the overexpression of tagged GLUT4 (207–209). However, GLUT4 overexpression can alter insulin sensitivity and kinase activities in an insulin resistance context, which limits its use in studies involving genetic or pharmacological manipulation with endogenous GLUT4. Significantly, recent research has made significant strides in understanding the mechanisms of GLUT4 translocation and localization. Tucker’s team developed antibodies that target the external surface epitopes of endogenous GLUT4, providing a robust method for detecting and quantifying transport-competent endogenous GLUT4 in the PM (210). James’ laboratory established an imaging-based assay to evaluate endogenous GLUT4 trafficking in cultured murine and human adipocytes (211). This innovative approach offers a more precise depiction of the localization and behavior of endogenous GLUT4 with heightened sensitivity, making it more suitable for refining insulin resistance models that necessitate physiological intervention. Meanwhile, it overcomes the limitations of traditional assays that depend on the overexpression of GLUT4 reporter constructs, which may confer protection against insulin resistance and exhibit resistance to genetic perturbations and induced insulin resistance treatment (211).

#### 3.3.3 Glycogen assay

*In vivo*, excessive glucose (after fully consumed by the body) can be converted into glycogen, which is stored in the liver and muscle cells as liver glycogen and muscle glycogen, respectively. Hepatic insulin resistance can increase the hepatic glucose production, decrease glycogen synthesis, and eventually raise blood glucose level (212, 213). Consequently, glycogen is used as an indicator for insulin resistance evaluation in liver and muscle cells *in vitro*, and the glycogen content in these cells can be directly measured using glycogen assay kits. For instance, one study explored the anti-insulin resistance activity of lrisin (a natural product) in 10 ng/ml TNF-α -induced HepG2 cells. The glycogen was detected after stimulating cells with 100 nM insulin, the reduction of glycogen in TNF-α-induced HepG2 and the increase of glycogen in lrisin-treated cells were used to evaluate insulin resistance model and anti-insulin resistance activity of lrisin, respectively (214). Moreover, Lei et al. induced insulin resistance in HepG2 cells by incubating them with 5000 nM insulin for 24h; subsequent glycogen content measurement revealed a significant decrease of glycogen synthesis in insulin resistance group (155). However, several studies have raised concerns about the reliability of glycogen content as a readout of insulin resistance. For example, it was found that, no obvious changes in the insulin-stimulated glycogen synthesis were exhibited by HepG2 cells, suggesting HepG2 cells may show a lack of sensitivity to the increase in glycogen synthesis induced by insulin stimulation (215). In adult skeletal muscle, it should be noted that, although there is no significant change in overall glycogen synthesis in response to insulin stimulation, the basal expression levels of key enzymes for glycogen synthesis, such as AKT and GSK3, are lower in L6 cells compared to C2C12 cells (66). These differences highlight the importance of considering cell type-specific factors when using glycogen as a readout for insulin resistance.

#### 3.3.4 Lipolysis markers

Lipolysis, the process whereby triglycerides in adipocytes are metabolized into glycerol and FFAs, is integral to the physiological modulation of insulin resistance and insulin signaling, and it is hormonally regulated by catecholamines, glucagon and cortisol (216). These hormones can activate protein kinase A (PKA) in a cAMP dependent manner, subsequently stimulating key enzymes such as hormone-sensitive lipase, thereby promoting adipocyte lipid breakdown and leading to the release of FFA or glycerol into the bloodstream (217, 218). In metabolic disorders (including obesity and type 2 diabetes), adipose tissue frequently exhibits elevated basal lipolysis, which leads to chronically high FFA levels or ectopic lipid deposition, and further impairing insulin signaling both in the liver and skeletal muscle (219). The accumulation of FFAs and their subsequent metabolites, such as diacylglycerol and ceramides, can inhibit IRS and disrupt the PI3K/Akt signaling pathway via activating protein kinase C (PKC), ultimately reducing insulin-mediated glucose uptake (220, 221). Therefore, numerous recent studies have employed lipolysis markers as the readouts of insulin sensitivity (218, 222–224). For example, Zhao et al. reported that metformin and resveratrol inhibit hypoxia-induced lipolysis by preserving PDE3B activity and blocking the cAMP/PKA/HSL signaling pathway, which prevents muscle insulin resistance by decreasing DAG deposition and suppressing PKCθ activation (222). Similarly, Jiang et al. showed that Astragaloside IV ameliorates insulin resistance in TNF-α-induced 3T3L1 adipocytes by dose-dependently suppressing lipolysis (224). In addition, lipolysis activity can be quantified by measuring the release of glycerol into the medium. Briefly, samples are cultured under conditions that regulate lipolysis, such as exposure to isoproterenol or insulin. The released glycerol is then quantified using either colorimetric or fluorescent detection methods (225).

Nevertheless, a recent clinical study suggests that lipolysis does not invariably impair insulin signaling, because the FFAs produced in response to metabolic demands can serve as crucial energy sources (220). Moreover, tissue sensitivity to the accumulation of FFAs is not entirely same certain tissues are more vulnerable to the detrimental effects of FFAs, whereas others demonstrate a higher tolerance (226). Although inhibition of lipolysis shows a beneficial effect on improving insulin sensitivity, excessive suppression of lipolysis could potentially disrupt metabolic homeostasis due to inadequate energy supply (219). Overall, strategies aimed at managing insulin resistance through lipolysis necessitate a nuanced approach that considers both systemic and localized metabolic effects.

#### 3.3.5 protein synthesis

There is no doubt that protein synthesis significantly contributes to the physiological modulation of insulin signaling and insulin resistance. Under normal insulin signaling conditions, activation of the insulin receptor triggers the IRS and PI3K-AKT signaling pathways, which facilitates glucose uptake, glycogen synthesis and protein synthesis (118). While in pathological conditions such as type 2 diabetes and hyperinsulinemia, chronic high insulin levels can lead to excessive mechanistic target of rapamycin complex 1 (mTORC1) activation, thereby increasing the burden of protein synthesis, disrupting ER homeostasis, as well as triggering the unfolded protein response. Initially, unfolded protein response (UPR) functions as a protective mechanism via suppressing protein synthesis to alleviate ER stress. However, persistent activation causes ER dysfunction and further exacerbates insulin resistance (227). Evidence have indicated that pretreating cells with protein synthesis inhibitors (such as cycloheximide), can alleviate insulin resistance caused by glucocorticoids (dexamethasone) or high-glucose environments, suggesting the significant role of protein synthesis load in insulin sensitivity (228). In recent years, the measurement of protein synthesis has been performed in studies associated with insulin resistance (229–231). Gao et al. found that major urinary protein 1 (MUP1) alleviates chemically induced ER stress and subsequent insulin resistance by inhibiting protein synthesis (232). Similarly, Marshall et al. demonstrated that the inhibition of mRNA synthesis with actinomycin D or 5,6-dichloro-1-beta-D-ribofuranosylbenzimidazole (DRB) fully protects adipocytes from glucose-induced insulin resistance (233). Methodologically, the quantitative protein synthesis can be assessed using experiments such as the [3H]-leucine incorporation assay. In this process, cells are incubated with radiolabelled leucine, followed by the precipitation of the protein and the removal of any unincorporated leucine. Subsequently, the degree to which the leucine has been incorporated into the newly synthesized protein is then quantified (225).

Nevertheless, the regulation of protein synthesis has a relatively limited impact on other components of the insulin signaling pathway, such as the expression and trafficking of IRS-1 or the GLUT4 transporter protein (234). This specificity can be also observed under high insulin concentrations, where protein synthesis is stimulated but has a minimal effect on glucose transporter proteins, leading to reduced insulin sensitivity rather than generalized insulin resistance (228, 230). Moreover, mRNA synthesis inhibitors (such as actinomycin D) partially prevent glucocorticoid-induced desensitization of the glucose transport system by inhibiting short-lived protein synthesis but do not significantly affect overall insulin responsiveness (235). Therefore, although protein synthesis regulation shows a beneficial role in alleviating ER burden and modulating insulin sensitivity, it fails to reflect a comprehensive state of cellular insulin resistance (232, 236).

#### 3.3.6 Insulin signal pathway indicators

**Figure 2:**
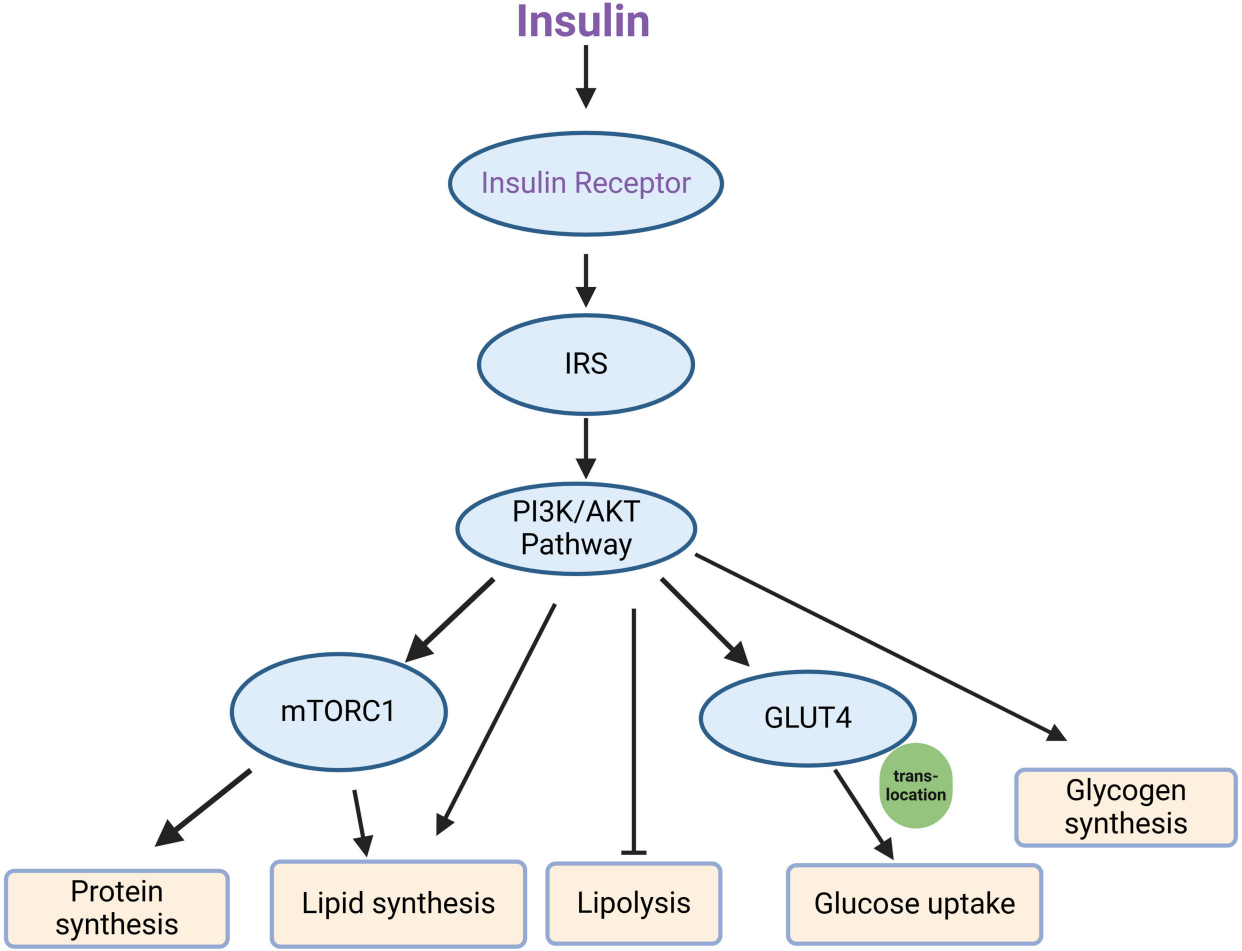
Key components of the canonical insulin signaling pathway. Upon insulin binding, the insulin receptor is activated, leading to the phosphorylation of IRS (insulin receptor substrate), which then engages the PI3K/AKT signaling pathway. This pathway subsequently activates several downstream effectors, including mTORC1 and GLUT4. mTORC1 mediates processes such as protein synthesis and lipid synthesis, while also inhibiting lipolysis. Simultaneously, GLUT4 translocates to the cell membrane, facilitating glucose uptake, and the pathway promotes glycogen synthesis.

Abnormalities within the insulin signaling pathway represent both the cause and consequence of insulin resistance. The functionality of this pathway is reflected in the expression levels and activities of its key components, including the insulin receptor, IRS, glucose transporters, and other associated signaling molecules. In particular, the phosphorylated status and activation of IRS downstream regulators are crucial indicators of pathway engagement and response to insulin stimulation (132, 237). Key signaling molecules in the canonical insulin signaling pathway, from the receptor to downstream effectors involved in glucose uptake, inhibition of lipolysis, protein synthesis and glycogen synthesis, include the insulin receptor, IRS, PI3K, AKT, and GLUT4 transporters (50, 51). The canonical insulin signaling pathway has been shown in **Figure 2**. The activation of these nodes is often assessed by phosphorylation of specific sites (such as IRS1 Ser 307, AKT Thr 308, and Ser 473), which are essential markers of pathway engagement upon insulin stimulation (237, 238).

However, relying solely on the detection of these molecular markers as indicators of insulin resistance, often assessed through western blot or qRT-PCR analysis, may not always be rigorous. Some studies on mechanisms of drug action measured the expression levels of these proteins but failed to evaluate more direct indicators of insulin resistance, such as glucose tolerance or insulin-stimulated glucose uptake. Thus, a more comprehensive assessment is need to accurately reflect the presence and severity of insulin resistance (239, 240).

There is also an ongoing discussion about defective signaling in insulin resistance, especially regarding receptor availability and pathway redundancy. The “spare receptor hypothesis” suggests that, in some insulin-resistant phenotypes, partial signaling through the available receptors may suffice for certain metabolic responses, even when phosphorylation of key signaling nodes is reduced (132, 241). This hypothesis suggests that not all insulin-resistant phenotypes can be attributed solely to diminished phosphorylation of critical signaling components, and that additional approaches to assess insulin sensitivity that go beyond phosphorylation status are nedded.

## 4 Conclusion

Cell culture, a fundamental tool in modern biological sciences, provides a crucial platform for exploring biological processes. It has been pivotal in studying the pathogenesis of diabetes and insulin resistance, as well as in identifying potential therapeutic interventions for these conditions. Therefore, the development and assessment of insulin resistance cell models are paramount, particularly for preliminary screening of anti-insulin resistance agents. Through comprehensive investigation and reflection, we recommend that several factors and details be carefully considered during the experimental process, including the main selection of cell lines, induction methods and evaluation metrics for insulin resistance (show in **Table 1**). Such considerations will ultimately aid in the clinical diagnosis and treatment of diseases linked to insulin resistance.

**Table 1:** Information of the representative methods for *in vitro* insulin resistance cell model.

Model	Context of Use	Cell Lines	Outcomes	Caveats	References
HCI	Simulating hyperinsulinemia to study insulin signaling defects. Commonly used at 100-1000 nM insulin for 24-48h.	HepG2, 3T3-L1, C2C12, SGBS	HCI triggers the preferential internalization and degradation of kinase-competent insulin receptors, resulting in a population of receptors with multiple functional abnormalities accumulated on the cell surface.	(1) Cannot fully replicate the multifactorial nature of insulin resistance *in vivo*; (2) May be temporary, with cells potentially reverting to their original insulin sensitivity levels upon cessation of the insulin treatment; (3) Fail to replicate physiological conditions.	(82, 103, 115, 130–133, 193, 242, 243)
HCG	Replicating the hyperglycemia observed in diabetic patients. Cells are exposed to 30-60 mM glucose for 24-48h.	HepG2, L6, 3T3-L1,	HCG induction can inflict cellular damage by compromising cell membrane integrity or hastening ROS-mediated stress responses, further impair mitochondrial function, resulting in cellular damage and apoptosis.	(1) Cause cell damage, called glucose toxicity; cell viability should be considered; (2) The used glucose concentration should be physiologically appropriate, particularly for drug screening and evaluation purposes.	(35, 139, 141, 144–146, 244–251)
FFAs	Simulating the obesity-related insulin resistance. PA and OA are typically used, at concentrations of 100 µM to 1,000 µM for 6-48h.	HepG2, 3T3-L1, C2C12, L6	Excessive FFAs can lead to dysfunction in gluconeogenesis and glycogen synthesis by increasing the level of intracellular phosphorylated glycogen synthase, down-regulating the phosphorylation of AKT, reducing the expression of glycogen synthase, and inhibiting the insulin signal pathway.	(1) Show cytotoxicity and cause cell damage, cell viability should also be considered; (2) The protective effect on low dose of FFA (such as OA or linoleate) should be noted when using mixed FFAs.	(33, 61, 150, 151, 160, 161, 180, 252–259)

Model, Name of the model used to simulate insulin resistance; Context of Use, Description of how and why the model is used, including typical experimental conditions (concentrations, exposure time); Cell Lines, Commonly used cell lines in previous studies; Outcomes: Key findings or results observed when the model is applied; Caveats: Potential caveats or challenges associated with the model; References: Citations supporting the use of the model and its outcomes.
